# Genetic Architecture of Winter Hardiness and Frost Tolerance in Triticale

**DOI:** 10.1371/journal.pone.0099848

**Published:** 2014-06-13

**Authors:** Wenxin Liu, Hans Peter Maurer, Guoliang Li, Matthew R. Tucker, Manje Gowda, Elmar A. Weissmann, Volker Hahn, Tobias Würschum

**Affiliations:** 1 Crop Genetics and Breeding Department, China Agricultural University, Beijing, China; 2 State Plant Breeding Institute, University of Hohenheim, Stuttgart, Germany; 3 ARC Centre of Excellence for Plant Cell Walls, University of Adelaide, Urrbrae, Australia; 4 Saatzucht Dr. Hege GbR Domäne Hohebuch, Waldenburg, Germany; Institute for Sustainable Agriculture (IAS-CSIC), Spain

## Abstract

Abiotic stress experienced by autumn-sown crops during winter is of great economic importance as it can have a severe negative impact on yield. In this study, we investigated the genetic architecture of winter hardiness and frost tolerance in triticale. To this end, we used a large mapping population of 647 DH lines phenotyped for both traits in combination with genome-wide marker data. Employing multiple-line cross QTL mapping, we identified nine main effect QTL for winter hardiness and frost tolerance of which six were overlapping between both traits. Three major QTL were identified on chromosomes 5A, 1B and 5R. In addition, an epistasis scan revealed the contribution of epistasis to the genetic architecture of winter hardiness and frost tolerance in triticale. Taken together, our results show that winter hardiness and frost tolerance are complex traits that can be improved by phenotypic selection, but also that genomic approaches hold potential for a knowledge-based improvement of these important traits in elite triticale germplasm.

## Introduction

Triticale (x *Triticosecale* Wittmack L.; 2n = 6x = 42) combines properties of its two parents, wheat and rye, and is primarily grown in the northern hemisphere. In Central Europe it is commonly planted as winter triticale in autumn, as varieties with a winter growth habit usually have a higher yield potential than varieties planted in spring. A prerequisite for these winter-type varieties is that they possess an adequate tolerance to endure the harsh conditions during winter in temperate zones. The capability of these plants to survive winter can be referred to as winter hardiness, involving among other factors freezing tolerance, desiccation, anoxia and disease resistance [1]. Of these, frost tolerance, i.e. the ability to survive freezing temperatures, has been considered as the major limiting factor in most regions. Owing to the economic importance, these two traits have been investigated for many decades and show complex genetic regulation [1]. Consequently, progress by breeders to improve these traits has been slow, which is further aggravated by the variable occurrence of this type of stress across years.

An additional complication when studying frost tolerance is that the trait can be affected by genes conferring low temperature tolerance as well as developmental genes with pleiotropic effects [2]. As reproductive meristems are more sensitive to low temperatures, small differences in developmental stage can affect the plants' ability to survive. As a consequence, genes controlling the transition to the reproductive phase by photoperiod (*Ppd*) or vernalization (*Vrn* genes) can also have an impact on frost tolerance [2]–[4]. To date, two major loci affecting frost tolerance have been identified in diploid and polyploid wheat and mapped to chromosome 5. *Frost Resistance-1* (*Fr-1*) maps close to *Vernalization-1* (*Vrn-1*) and has therefore been suggested to present a pleiotropic effect of this major vernalization gene [2], [5], [6]. *Vrn-1* encodes a MADS box transcription factor similar to the Arabidopsis meristem identity gene *AP1* and is upregulated during vernalization [7]. The second locus underlying frost tolerance, *Fr-2*, has been mapped approximately 30 cM proximal to *Vrn-1* [8]–[10] and the molecular characterization has revealed a cluster of multiple *Cbf* (*C-repeat Binding Factor*) genes at this locus that are upregulated by low temperatures [11], [12]. In wheat, copy number variation at the *Fr-2* locus was recently reported to be associated with frost tolerance [13]. For both *Fr* QTL, homoeologous loci have been reported on the A, B and D genomes of wheat [14]–[16]. In rye, twelve members of the *Cbf* family have also been assigned to the long arm of chromosome 5R [17] and some have recently been reported to be significantly associated with frost tolerance [18]. In addition to the two major *Fr-1* and *Fr-2* loci, the complex genetic architecture underlying this trait suggests the presence of other genes with small effects on frost tolerance [19].

The aim of this study was to dissect the genetic basis underlying winter hardiness and frost tolerance in triticale, identifying targets for knowledge-based improvement of these two agronomically important traits. To this end, we employed multiple-line cross QTL mapping [20] based on a large triticale mapping population with 647 DH lines derived from four families. In particular, the objectives were to (i) investigate the phenotypic inheritance of the two traits, (ii) detect main effect QTL and (iii) assess the contribution of epistasis.

## Materials and Methods

### Plant material, field trials and phenotypic data

The plant material and field trials underlying this study have been described previously [21], [22]. In brief, the mapping population consisted of 647 doubled haploid (DH) [23], [24] triticale (x *Triticosecale* Wittmack L.) lines derived from four families, DH06 (131), DH07 (120), EAW74 (200), and EAW78 (196) that have been described by Alheit et al. [25], [26]. The DH lines were grown in partially replicated designs [27] including common checks with 960 plots per location and analyzed by alpha analysis of variance. In 2012 two locations (Eckartsweier and Hohebuch) showed severe frost without snow coverage. In Eckartsweier the average temperature in February was −2.0°C and the minimum temperature was −17.8°C while in Hohebuch the average temperature was −2.2°C and the minimum temperature −17.7°C. At both locations winter hardiness was scored as the appearance of the plants at the end of winter (beginning of April) on a scale between 1 (no damage) to 9 (maximum damage, i.e., no plant survived). In addition, the direct effect of frost was scored at one location (Eckartsweier) after the frost had occurred (i.e. frost damage at the end of February), with a score between 1 (no damage) to 9 (maximum damage). For all lines the Best Linear Unbiased Estimates (BLUEs) were determined. All mixed model calculations were performed using the software ASReml 3.0 [28].

### Multiple-line cross QTL mapping

The DH lines were genotyped with DArT markers and QTL mapping was done based on the integrated consensus linkage map described by Alheit et al. [25]. For QTL mapping, an additive genetic model was chosen and a joint analysis was performed with a model assuming specific QTL effects for every family [20], [29] as described in detail by Steinhoff et al. [30]. In brief, the multiple-line cross QTL mapping model was: 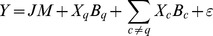
where Y was a *N*×1 column vector of the BLUE values of phenotypic data of *N* progenies coming from *P* families. J was a *N*×*P* matrix whose elements were 1 or 0 according to whether or not individual *i* belonged to family *p* and *M* was a *P*×1 vector of family specific means. X_q_ (X_c_) a *N*×*P* matrix containing the expected number (ranging from 0 to 2) of allele *k* for each individual in family *p* at QTL *q* (cofactor *c*), and B_q_ (B_c_) was a *P*×1 vector of the expected allele substitution effects of QTL *q* (cofactor *c*) in family *p*. ε was the vector of the residuals.

Cofactor selection was performed using PROC GLMSELECT implemented in the statistical software SAS [31]. The presence of a putative QTL in an interval was tested using a likelihood-ratio test with the statistical software R [32]. LOD-thresholds of 4.71 for winter hardiness and 4.66 for frost tolerance were used corresponding to an experiment-wise type I error of *P*<0.10, based on 2000 permutations [33]. The proportion of genotypic variance explained by the detected QTL was estimated as *R^2^_adj_*/*h^2^* [34]. The support interval of a QTL was defined as a LOD fall-off of 1.0 expressed as position on the chromosome in centimorgans (cM) [35] and cofactors were excluded within a distance to the marker interval under consideration smaller than 10 cM. QTL were declared as overlapping between winter hardiness and frost tolerance if they fell within an arbitrarily defined 10 cM interval surrounding the QTL. Fivefold cross-validation was done as described previously [36], [37].

The epistasis scan for pairwise interactions was done with the model described above which was extended by the term X*_q′_*B*_q′_* for the second locus and the interaction term X*_qq′_*B*_qq′_* between the two loci *q* and *q′*. We used an α-level of 0.05 and followed the suggestion of Holland et al. [38] dividing the α-level by the number of possible independent pairwise interactions between chromosome regions, assuming two separate regions per chromosome (*P*<5.3e-5). The circular plots illustrating the epistatic interactions were created with Circos [39].

## Results

**Figure 1:**
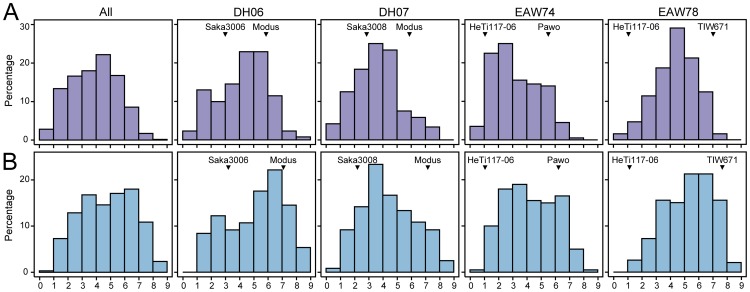
Histograms of the phenotypic values. Shown for (**a**) winter hardiness and (**b**) frost tolerance for the entire population (All) and for each of the four families (DH06, DH07, EAW74, EAW78). The arrowheads indicate the phenotypic values of the respective parents.

In the mapping population with 647 DH lines, we observed significant (*P*<0.01) genotypic variances 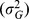 for both winter hardiness and frost tolerance (Table 1). For winter hardiness, which was evaluated at two locations, the genotype by environment interaction variance 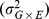 could be estimated. This was also found to be significant and the ratio of genotypic variance to genotype by environment interaction variance was 9∶1. For both traits the observed phenotypic values covered the full range from highly susceptible to fully tolerant (Table 1, Figure 1). The heritability was high with 0.87 for winter hardiness and the repeatability was 0.91 for frost tolerance, while the the phenotypic correlation between both traits was 0.88 (P<0.01). The phenotypic values of the parents differed to varying degrees ranging from Δ2.9 to 5.9 for winter hardiness and from Δ3.9 to 6.6 for frost tolerance (Figure 1). The trait distributions approximately followed a normal distribution and in each family DH lines transgressed the respective parents (Figure 1). Taken together, these results illustrate that the mapping population is well suited to study the genetics underlying winter hardiness and frost tolerance in triticale.

**Table 1:** Summary statistics for winter hardiness and frost tolerance.

	Winter hardiness	Frost tolerance
Min	0.9	0.4
Mean	3.9	4.8
Max	8.1	8.9
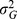	3.00**	2.36**
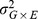	0.33**	n.a.
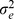	0.81	0.34
*h* ^2^	0.87	0.91

Genotypic variance 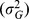, genotype by location interaction variance 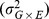, error variance 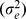, and heritability (*h^2^*).

** significant at the 0.01 probability level.

**Figure 2:**
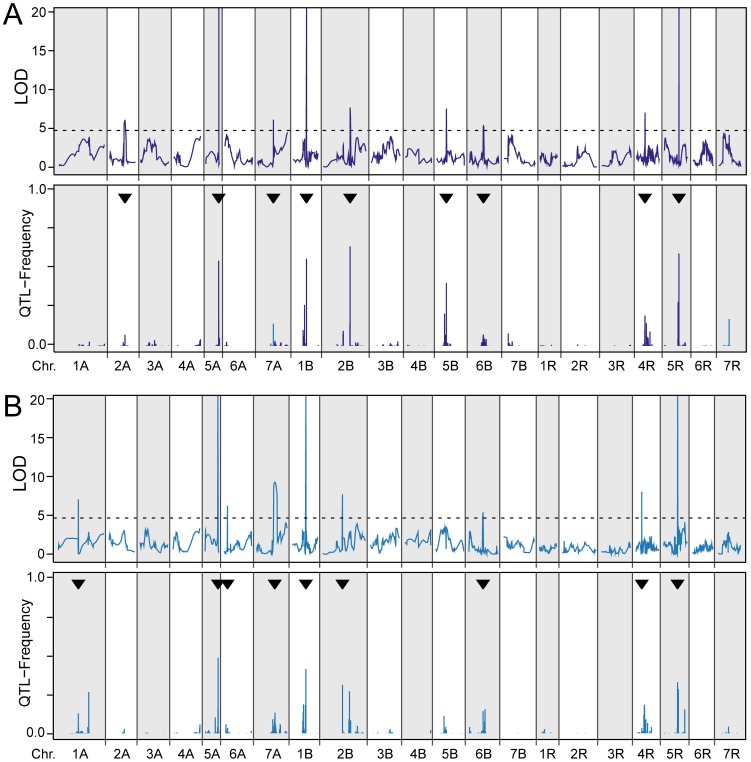
Results from QTL mapping. QTL LOD scores and QTL frequency distributions from fivefold cross-validation for the QTL detected for (**a**) winter hardiness and (**b**) frost tolerance. The arrowheads indicate the QTL detected with the full data set and the horizontal dashed line the significance threshold.

Multiple-line cross QTL mapping revealed 9 QTL for winter hardiness as well as for frost tolerance (Table 2, 3, Figure 2) of which 6 were overlapping between both traits. The detected QTL explained 63.0 and 59.8 percent of the genotypic variance of winter hardiness and frost tolerance, respectively. The proportion of genotypic variance explained by single QTL ranged from 2.0 to 24.1 percent for winter hardiness and from 1.9 to 16.3 percent for frost tolerance (Table 3). For winter hardiness as well as for frost tolerance three major QTL explaining more than 10 percent of genotypic variance were detected on chromosomes 5A, 1B and 5R. Interestingly, the major QTL on chromosome 1B appeared to segregate in all four families while family EAW74 did not segregate for the QTL on 5R but was the only family segregating for the QTL on chromosome 5A. The single family analysis revealed one more major QTL for frost tolerance located on chromosome 6A, segregating in family DH07.

**Table 2:** Results of QTL mapping and fivefold cross-validation.

	Winter hardiness	Frost tolerance
QTL_DS_	9	9
*p*_G-DS_	63.0	59.8
QTL_ES_	8.5	8.8
*p*_G-ES_	52.5	52.0
*p*_G-TS_	31.3	30.0
Relative bias	40.4	42.3

Number of detected QTL (QTL_DS_), proportion of genotypic variance (%) explained by the detected QTL across all families in the data set (*p*_G-DS_), number of QTL (QTL_ES_) and proportion of genotypic variance averaged over estimation sets (*p*_G-ES_) and averaged over test sets (*p*_G-TS_), and relative bias (%) in the estimation of *p*_G_.

**Table 3:** QTL detected for winter hardiness and frost tolerance.

Chromosome	Position in cM [support interval]	*p*_G_	*p_G_* DH06	*p_G_* DH07	*p_G_* EAW74	*p_G_* EAW78
*Winter hardiness*						
2A	62.1 [58.0–63.8]	2.2	0.6	3.6	0.3	0.2
5A	52.7 [51.6–52.9]	14.1	1.2	0.2	40.2	0.0
7A	63.6 [63.5–63.7]	2.3	0.0	0.5	3.1	3.3
1B	54.1 [53.2–54.3]	12.5	14.4	17.4	7.6	18.8
2B	107.4 [107.0–108.1]	2.9	4.5	3.4	3.1	4.1
5B	39.9 [39.3–40.7]	2.8	2.1	2.2	5.3	1.3
6B	56.8 [55.3–58.7]	2.0	1.5	6.8	0.7	0.2
4R	35.7 [35.3–36.1]	2.6	0.0	0.2	4.8	2.3
5R	58.9 [58.6–58.9]	24.1	44.8	20.4	3.4	29.8
A genome		18.6	1.8	4.3	43.6	3.5
B genome		20.2	22.5	29.8	16.7	24.4
R genome		26.7	44.8	20.6	8.2	32.1
*Frost tolerance*						
1A	80.1 [80.0–80.4]	2.6	0.0	8.3	1.4	1.5
5A	52.5 [51.6–52.9]	12.4	2.1	0.5	40.2	0.3
6A	14.5 [14.0–14.6]	2.2	0.0	13.1	0.0	0.2
7A	71.6 [66.8–79.3]	3.4	0.4	4.1	3.8	1.9
1B	54.1 [53.2–54.3]	16.3	19.0	15.4	5.1	25.3
2B	79.3 [78.5–79.3]	2.9	5.6	3.4	4.3	4.1
6B	57.8 [55.3–58.7]	1.9	1.6	2.5	3.1	0.6
4R	25.3 [25.1–25.3]	3.0	0.8	4.5	6.9	0.0
5R	55.6 [55.4–56.2]	14.7	31.7	9.0	1.5	18.2
A genome		20.6	2.5	26.0	45.4	3.9
B genome		21.1	26.2	21.3	12.5	30.0
R genome		17.7	32.5	13.5	8.4	18.2

Chromosome, position with support interval with a LOD fall off of 1.0 and proportion of genotypic variance explained by the QTL (*p_G_* in %) in the entire population and in each of the families, and the summary for the three genomes.

We used fivefold cross-validation to obtain less biased estimates of the proportion of genotypic variance (*p_G_*) explained by the QTL (Table 2). The cross-validated *p_G_* was 31.1 and 30.0 percent for winter hardiness and frost tolerance, respectively. The QTL frequency distributions revealed that some QTL including the three major QTL were detected in a high number of runs further substantiating their importance (Figure 2).

**Figure 3:**
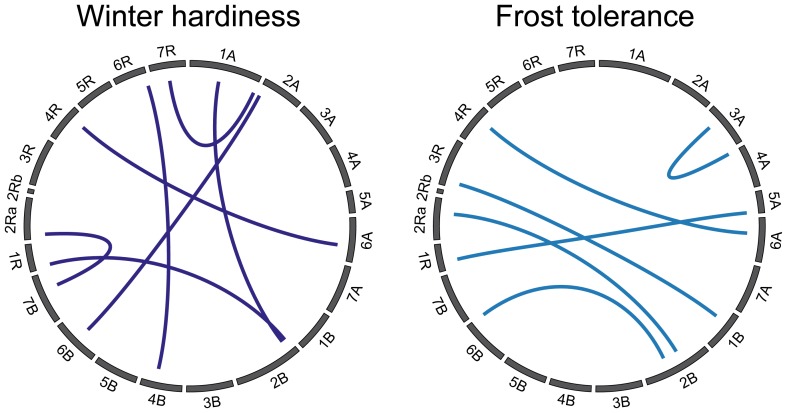
Epistatic QTL for winter hardiness and frost tolerance. Circular plots illustrating interactions among loci.

The full 2-dimensional epistasis scan revealed seven epistatic QTL for winter hardiness and six for frost tolerance (Figure 3). Interestingly, the epistatic QTL for frost tolerance between chromosomes 5A and 1R involves the same chromosomal region on chromosome 5A (at ∼53 cM) that was identified as major QTL. The contribution of these epistatic QTL to the genotypic variance was small and ranged between 0.1 and 3.5 percent for winter hardiness and between 0.9 and 4.1 percent for frost tolerance.

## Discussion

Frost tolerance is one of the most important abiotic stresses. In both hemispheres it is a factor limiting the geographic distribution of crops but can also have severe effects on crop production potentially resulting in complete losses. The higher yield makes winter varieties attractive but requires breeding for increased winter hardiness and frost tolerance to ensure survival of the plants during winter. Whereas winter hardiness and frost tolerance have been studied in wheat and rye, little is known about the inheritance of both traits in triticale. The aim of this study therefore was to investigate the genetic architecture of winter hardiness and frost tolerance in triticale.

### Phenotypic evaluation

Triticale (AABBRR) contains both wheat (AABB) and rye (RR) genomes, with rye being an extremely frost tolerant species among small grain cereals [40], [41]. Although triticale is less frost tolerant than rye, we observed significant genotypic variances for winter hardiness and frost tolerance in the mapping population. The estimated genotypic values covered the full range from highly tolerant to fully susceptible illustrating the potential to improve these traits by breeding. The trait distributions highlight the quantitative nature of the traits, suggesting a contribution of many small-effect QTL but not excluding the presence of few major effect QTL. We observed substantial differences between the parents for each family. The transgressive segregation, however, suggests that the less tolerant parent carried favourable alleles not present in the better parent. Such positive alleles can be combined in the progeny resulting in lines that are superior to the parental lines, illustrating the potential of phenotypic selection to improve these traits in triticale. Consequently, crosses including the most frost tolerant lines identified here appears as a promising strategy to further increase the level of frost tolerance in elite triticale germplasm. The high correlation between winter hardiness and frost tolerance confirms frost tolerance as a major factor contributing to the plants' survival during winter.

A major limitation, however, for the conventional improvement of winter hardiness and even more so of frost tolerance is the wide variation in occurrence of these stresses across years. Semi-controlled tests have recently been shown to correlate well with results from field trials in durum wheat and thus offer a possibility to screen for frost tolerance irrespective of the snow coverage on the field [42]. Another option is the identification of QTL for marker-assisted selection [43]. The phenotypic data from the field trials in the year 2012 with its exceptionally strong frost in the absence of snow offers an excellent basis for the detection of QTL underlying winter hardiness and frost tolerance in triticale.

### Detection of main effect QTL

We identified a total of nine QTL for winter hardiness and frost tolerance of which six were detected for both traits. The high number of overlapping QTL detected for winter hardiness and frost tolerance further substantiates frost tolerance as a major contributor to winter survival. Among the identified QTL, three explained more than 10 percent of the genotypic variance for both traits and can be considered as major QTL (Table 3). One of these major QTL is located on chromosome 5A, the chromosome harbouring the two *Frost Resistance* (*Fr*) loci in wheat [1], [10]. The rather distal location of the identified QTL suggests that it may be *Fr-A1*/*Vrn-A1*. While *Fr-1* was initially thought to be an independent locus closely linked to *Vrn-1*, it is now believed to be one and the same locus, with *Vrn-1* exhibiting a pleiotropic effect on frost tolerance [2]–[4]. *Vrn-1* expression and the subsequent transition to the reproductive stage thereby negatively affect the induction of the *CBF* and other cold-induced genes [6], [44]. The QTL detected on chromosome 5R may be the rye homoeologue of *Vrn-1* (called *Sp1*) [45] which similar to the QTL identified here, maps roughly to the middle of chromosome 5R. Alternatively, this QTL contributed by the rye genome may represent the *CBF* cluster found to be associated with frost tolerance in rye [18]. However, the verification of the genes underlying the identified QTL as well as the molecular basis of the major QTL identified on chromosome 1B requires further research. Our findings illustrate that all genomes contribute to winter hardiness and frost tolerance in triticale. Interestingly, more of the detected QTL are derived from the wheat A and B genomes and not from the rye genome (Table 3), despite rye being the more frost tolerant species [41]. While primary triticale has been obtained by crosses between durum wheat with low winter hardiness and rye, secondary triticale has resulted from backcrosses with wheat, potentially explaining the source of the winter hardiness and frost tolerance QTL on the A and B genomes. In addition, phenotypic evaluation of octoploid and hexaploid triticale has revealed that their cold hardiness levels were always more similar to their wheat parents suggesting a suppression of the excellent rye cold hardiness in triticale, either by suppression of the responsible rye genes or by a dilution of their effects due to the polyploid nature of triticale [40]. Alternatively, important frost tolerance QTL alleles on the rye genome may be fixed in all six parents and may thus not be segregating in the mapping population. It must be noted that only segregating QTL will contribute to the phenotypic variation and can be detected, whereas fixed QTL cannot be detected but may provide the baseline level of frost tolerance present in all lines.

Despite the large mapping population which warrants a high QTL detection power, the cross-validated proportion of genotypic variance explained by the detected QTL amounted to only approximately 30 percent. This is likely due to the quantitative nature of the traits with many small-effect QTL that remained undetected. A recent study on frost tolerance in wheat has shown the potential of genomic selection to improve this trait [19]. Genomic selection also considers small effect QTL and the achieved prediction accuracy was higher as compared to that obtained with detected QTL. Taken together, our results show that marker-assisted selection based on the QTL detected by MC-QTL mapping or alternatively genomic selection, hold potential for a knowledge-based improvement of winter hardiness and frost tolerance in triticale.

### Contribution of epistasis

Epistasis refers to interactions between the alleles at two or more genetic loci in the genome [46] and has recently been shown to contribute to the genetic architecture of complex traits in different crops including maize, wheat and rapeseed [47]–[51]. Interactions among key factors underlying frost tolerance are present on the molecular level [1] suggesting that epistasis could contribute to the genetics of this trait. One significant epistatic interaction has been reported for the candidate gene association mapping study on frost tolerance in rye [18] and a number of epistatic interactions for the genome-wide association mapping approach in wheat [19]. Here, we identified seven epistatic QTL for winter hardiness and six for frost tolerance of which one might be identical in both traits (Chr 6A - 4R; Figure 3). Interestingly, one epistatic interaction involved the major QTL identified on chromosome 5A suggesting that this locus, which might be *Vrn-1*, is also involved in epistatic interactions. Our results thus corroborate previous findings from wheat and rye and show the contribution of epistasis to the genetic architecture of winter hardiness and frost tolerance in triticale.

### Conclusions

In this study, we employed multiple-line cross QTL mapping based on a large DH mapping population to dissect the genetic architecture of winter hardiness and frost tolerance in triticale. We identified main effect QTL on all three genomes and three major effect QTL, some of which potentially correspond to known frost tolerance loci. In addition, our results reveal the contribution of epistasis to both traits. In summary, winter hardiness and frost tolerance in triticale are complex traits that can be improved by phenotypic selection but also hold the potential for a knowledge-based selection of superior lines.

## References

[1] Galiba G, Vágújfalvi A, Li C, Solté A, Dubcovsky J (2009) Regulatory genes involved in the determination of frost tolerance in temperate cereals. Plant Science 176: 12–19.

[2] Limin AE, Fowler DB (2006) Low-temperature tolerance and genetic potential in wheat (Triticum aestivum L.): response to photoperiod, vernalization, and plant development. Planta 224: 360–366. 16440213 10.1007/s00425-006-0219-y

[3] Limin AE, Fowler DB (2002) Developmental traits affecting low-temperature tolerance response in near-isogenic lines for the vernalization locus Vrn-A1 in wheat (Triticum aestivum L. em Thell). Annals of Botany 89: 579–585. 12099532 10.1093/aob/mcf102 PMC4233904

[4] Mahfoozi S, Limin AE, Fowler DB (2001) Developmental regulation of low-temperature tolerance in winter wheat. Annals of Botany 87: 751–757.

[5] Sutka J, Snape JW (1989) Location of a gene for frost resistance on chromosome 5A of wheat. Euphytica 42: 41–44.

[6] Dhillon T, Pearce S, Stockinger EJ, Distelfeld A, Li C, et al (2010) Regulation of freezing tolerance and flowering in temperate cereals: The VRN-1 connection. Plant Physiology 153: 1846–1858. 20571115 10.1104/pp.110.159079 PMC2923912

[7] Yan L, Loukoianov A, Tranquilli G, Helguera M, Fahima T, et al (2003) Positional cloning of wheat vernalization gene VRN1. Proc Natl Acad Sci USA 100: 6263–6268. 12730378 10.1073/pnas.0937399100 PMC156360

[8] Vágújfalvi A, Crosatti C, Galiba G, Dubcovsky J, Cattivelli L (2000) Two loci on wheat chromosome 5A regulate the differential cold-dependent expression of the cor14b gene in frost-tolerant and frost-sensitive genotypes. Molecular and General Genetics 263: 194–200. 10778737 10.1007/s004380051160

[9] Vágújfalvi A, Galiba G, Cattivelli L, Dubcovsky J (2003) The cold-regulated transcriptional activator Cbf3 is linked to the frost-tolerance locus Fr-A2 on wheat chromosome 5A. Molecular and General Genetics 269: 60–67. 10.1007/s00438-003-0806-6 PMC4743881 12715154

[10] Båga M, Chodaparambil SV, Limin AE, Pecar M, Folwer DB, et al (2007) Identification of quantitative trait loci and associated candidate genes for low-temperature tolerance in cold-hardy winter wheat. Funct Integr Genomics 7: 53–68. 16775685 10.1007/s10142-006-0030-7

[11] Vágújfalvi A, Aprile A, Miller A, Dubcovsky J, Delugu G, et al (2005) The expression of several Cbf genes at the Fr-A2 locus is linked to frost resistance in wheat. Mol Genet Genomics 274: 506–514. 16200412 10.1007/s00438-005-0047-y

[12] Miller AK, Galiba G, Dubcovsky J (2006) A cluster of 11 CBF transcription factors is located at the frost tolerance locus Fr-A^m^2 in Triticum monococcum. Mol Genet Genomics 275: 193–203. 16362370 10.1007/s00438-005-0076-6

[13] Dhillon T, Stockinger EJ (2013) Cbf14 copy number variation in the A, B, and D genomes of diploid and polyploid wheat. Theor Appl Genet 126: 2777–2789. 23918064 10.1007/s00122-013-2171-0

[14] Sutka J (2001) Genes for frost resistance in wheat. Euphytica 119: 167–172.

[15] Tóth B, Galiba G, Fehér E, Sutka J, Snape JW (2003) Mapping genes affecting flowering time and frost resistance on chromosome 5B of wheat. Theor Appl Genet 107: 509–514. 12734655 10.1007/s00122-003-1275-3

[16] Snape JW, Semikhodskii A, Fish L, Sarma RN, Quarrie SA, et al (1997) Mapping of frost tolerance loci in wheat and comparative mapping with other cereals. Acta Agronomica Hungarica 45: 268–270.

[17] Campoli C, Matus-Cadiz MA, Pozniak CJ, Cattivelli L, Fowler DB (2009) Comparative expression of Cbf genes in the Triticeae under different acclimation induction temperatures. Mol Genet Genomics 282: 141–152. 19421778 10.1007/s00438-009-0451-9 PMC2757611

[18] Li Y, Böck A, Haseneyer G, Korzun V, Wilde P, et al (2011) Association analysis of frost tolerance in rye using candidate genes and phenotypic data from controlled, semi-controlled, and field phenotyping platforms. BMC Plant Biol 11: 146. 22032693 10.1186/1471-2229-11-146 PMC3228716

[19] Zhao Y, Gowda M, Würschum T, Longin CFH, Korzun V, et al (2013) Dissecting the genetic architecture of frost tolerance in Central European winter wheat. J Exp Bot 64: 4453–4460. 24006418 10.1093/jxb/ert259 PMC3808325

[20] Blanc G, Charcosset A, Mangin B, Gallais A, Moreau L (2006) Connected populations for detecting quantitative trait loci and testing for epistasis: An application in maize. Theor Appl Genet 113: 206–224. 16791688 10.1007/s00122-006-0287-1

[21] Busemeyer L, Ruckelshausen A, Möller K, Melchinger AE, Alheit KV, et al (2013) Precision phenotyping of biomass accumulation in triticale reveals temporal genetic patterns of regulation. Sci Rep 3: 2442. 23942574 10.1038/srep02442 PMC3743059

[22] Alheit KV, Busemeyer L, Liu W, Maurer HP, Gowda M, et al (2014) Multiple-line cross QTL mapping for biomass yield and plant height in triticale (× *Triticosecale* Wittmack). Theor Appl Genet 127: 251–260. 24173688 10.1007/s00122-013-2214-6

[23] Würschum T, Tucker MR, Reif JC, Maurer HP (2012) Improved efficiency of doubled haploid generation in hexaploid triticale by in vitro chromosome doubling. BMC Plant Biology 12: 109. 22809089 10.1186/1471-2229-12-109 PMC3443072

[24] Würschum T, Tucker MR, Maurer HP (2013) Stress treatments influence efficiency of microspore embryogenesis and green plant regeneration in hexaploid triticale (× Triticosecale Wittmack L.). In Vitro Cell Dev Biol - Plant 50: 143–148.

[25] Alheit KV, Reif JC, Maurer HP, Hahn V, Weissmann EA, et al (2011) Detection of segregation distortion loci in triticale (× Triticosecale Wittmack) based on a high-density DArT marker consensus genetic linkage map. BMC Genomics 12: 380. 21798064 10.1186/1471-2164-12-380 PMC3156787

[26] Alheit KV, Maurer HP, Reif JC, Tucker MR, Hahn V, et al (2012) Genome-wide evaluation of genetic diversity and linkage disequilibrium in winter and spring triticale (x Triticosecale Wittmack). BMC Genomics 13: 235. 22691168 10.1186/1471-2164-13-235 PMC3464613

[27] Williams E, Piepho H-P, Whitaker D (2011) Augmented p-rep designs. Biometrical Journal 53: 19–27. 21259306 10.1002/bimj.201000102

[28] Gilmour AR, Gogel BG, Cullis BR, Thompson R (2009) ASReml user guide release 3.0. VSN International Ltd, Hemel Hempstead, HP1 1ES, UK.

[29] Würschum T, Liu W, Gowda M, Maurer HP, Fischer S, et al (2012) Comparison of biometrical models for joint linkage association mapping. Heredity 108: 332–340. 21878984 10.1038/hdy.2011.78 PMC3282402

[30] Steinhoff J, Liu W, Maurer HP, Würschum T, Longin CFH, et al (2011) Multiple-line cross quantitative trait locus mapping in european elite maize. Crop Sci 51: 2505–2516.

[31] SAS Institute (2008) SAS/STAT 9.2 User's guide., Cary NC.

[32] R Development Core Team (2010) R: a language and environment for statistical computing, R foundation for statistical computing. http://www.R-project.org.

[33] Doerge RW, Churchill GA (1996) Permutation tests for multiple loci affecting a quantitative character. Genetics 142: 285–294. 8770605 10.1093/genetics/142.1.285 PMC1206957

[34] Utz HF, Melchinger AE, Schön CC (2000) Bias and sampling error of the estimated proportion of genotypic variance explained by quantitative trait loci determined from experimental data in maize using cross validation and validation with independent samples. Genetics 154: 1839–1849. 10866652 PMC1461020

[35] Lander ES, Botstein S (1989) Mapping mendelian factors underlying quantitative traits using RFLP linkage maps. Genetics 121: 185–199. 2563713 10.1093/genetics/121.1.185 PMC1203601

[36] Liu W, Maurer HP, Reif JC, Melchinger AE, Utz HF, et al (2013) Optimum design of family structure and allocation of resources in association mapping with lines from multiple crosses. Heredity 110: 71–79. 23047199 10.1038/hdy.2012.63 PMC3522231

[37] Würschum T, Kraft T (2014) Cross-validation in association mapping and its relevance for the estimation of QTL parameters of complex traits. Heredity 112: 463–468. 24326292 10.1038/hdy.2013.126 PMC3966130

[38] Holland JB, Portyanko VA, Hoffmann DL, Lee M (2002) Genomic regions controlling vernalization and photoperiod responses in oat. Theor Appl Genet 105: 113–126. 12582569 10.1007/s00122-001-0845-5

[39] Krzywinski M, Schein J, Birol I, Connors J, Gascoyne R, et al (2009) Circos: An information aesthetic for comparative genomics. Genome Res 19: 1639–1645. 19541911 10.1101/gr.092759.109 PMC2752132

[40] Limin AE, Dvorak J, Fowler DB (1985) Cold hardiness in hexaploid triticale. Can J Plant Sci 65: 487–490.

[41] Fowler DB (2008) Cold acclimation threshold induction temperatures in cereals. Crop Sci 48: 1147–1154.

[42] Sieber AN, Würschum T, Longin CFH (2014) Evaluation of a semi-controlled test as a selection tool for frost tolerance in durum wheat (Triticum durum). Plant Breeding doi:10.1111/pbr.12181.

[43] Würschum T (2012) Mapping QTL for Agronomic Traits in Breeding Populations. Theor Appl Genet 125: 201–210. 22614179 10.1007/s00122-012-1887-6

[44] Pearce S, Zhu J, Boldizsár Á, Vágújfalvi A, Burke A, et al (2013) Large deletions in the CBF gene cluster at the Fr-B2 locus are associated with reduced frost tolerance in wheat. Theor Appl Genet 126: 2683–2697. 23884601 10.1007/s00122-013-2165-y PMC4779059

[45] Plaschke J, Börner A, Xie DX, Koebner RMD, Schelgel R, et al (1993) RFLP mapping of gene saffecting plant height and growth habit in rye. Theor Appl Genet 85: 1049–1054. 24196157 10.1007/BF00215046

[46] Carlborg Ö, Haley CS (2004) Epistasis: Too often neglected in complex trait studies? Nat Rev Genet 5: 618–625. 15266344 10.1038/nrg1407

[47] Buckler ES, Holland JB, Bradbury PJ, Acharya CB, Brown PJ, et al (2009) The genetic architecture of maize flowering time. Science 325: 714–718. 19661422 10.1126/science.1174276

[48] Reif JC, Maurer HP, Korzun V, Ebmeyer E, Miedaner T, et al (2011) Mapping QTLs with main and epistatic effects underlying grain yield and heading time in soft winter wheat. Theor Appl Genet 123: 283–292. 21476040 10.1007/s00122-011-1583-y

[49] Liu W, Reif JC, Ranc N, Porta GD, Würschum T (2012) Comparison of biometrical approaches for QTL detection in multiple segregating families. Theor Appl Genet 125: 987–998. 22618736 10.1007/s00122-012-1889-4

[50] Steinhoff J, Liu W, Reif JC, Porta GD, Ranc N, et al (2012) Detection of QTL for flowering time in multiple families of elite maize. Theor Appl Genet 125: 1539–1551. 22801873 10.1007/s00122-012-1933-4

[51] Würschum T, Maurer HP, Dreyer F, Reif JC (2013) Effect of inter- and intragenic epistasis on the heritability of oil content in rapeseed (Brassica napus L.). Theor Appl Genet 126: 435–441. 23052025 10.1007/s00122-012-1991-7

